# Imageology features of different types of multifocal choroiditis

**DOI:** 10.1186/s12886-019-1045-x

**Published:** 2019-02-01

**Authors:** Juanjuan Li, Yunpeng Li, Hua Li, Liwei Zhang

**Affiliations:** 1 Department of Ophthalmology, The Second People’s Hospital of Yunnan Province, No.176 Qingnian Rd, Kunming, 650021 Yunnan People’s Republic of China; 2 Department of Anti-drug, Yunnan Police Officer Academy, Kunming, 6500223 Yunnan China

**Keywords:** Choroiditis diagnosis, Fluorescein angiography, Tomography, optical examination

## Abstract

**Background:**

Multifocal choroiditis (MFC) is multi-inflammatory lesions that occur in the retinal pigment epithelium (RPE) and the choriocapillaris. Optical examinations are the major diagnostic methods to diagnose the disease.

**Objective:**

To examine patients with different types of MFC by multiple imageological methods. To summarize the imageology features of different types of MFC to make a medical examination guideline for clinician practices.

**Method:**

All of the patients who included in the study received examinations of fundus color photography, infrared fundus photography, fundus auto fluorescence (FAF), fluorescein fundus angiography (FFA), and optical coherence tomography (OCT), respectively. Finally, imageology features of different types of multifocal choroiditis were summarized.

**Results:**

A total of 51 eyes from 28 patients with diagnosed MFC were included in the study. These patients consisted of 10 males and 18 females aged from 31 to 49 (mean age: 41.5 ± 0.8). 23 patients had MFC on both eye whilst 5 had monocular disease. The MFC lesions were classified as active inflammatory lesions, inactive inflammatory lesions, inflammatory lesions secondary active choroidal neovascularization (CNV) and inflammatory lesions secondary inactive CNV according to literature reports and comprehensive fundus imaging examinations.

**Conclusion:**

Examinations via fundus color photography, infrared fundus photography, FAF, FFA and OCT indicate typical imageological signals of different types of MFC. These imageology tests can greatly assist the clinicians to identify the MFC and provide proper therapies.

## Background

Multifocal choroiditis (MFC) is an inflammatory disorder characterized by uveitis and multiple lesion that occurs in the retinal pigment epithelium (RPE) and the choroidal capillary layer [1]. Symptoms of MFC include blurry vision with or without sensitivity to light. Some patients suffer from floaters, blind spots and mild eye discomfort [1, 2]. MFC occurs spontaneously and the cause is currently not known. Some hypothesized that a bacterial or viral infection may trigger the immune response that results in the inflammation [3], it is controversial though [4]. The precise pathogenesis is needed to be studied. Current treatment to MFC includes periocular or systemic corticosteroids application [5]. These treatments are managed to alleviate the symptoms, but not offering a permanent cure. Immunosuppression agents like cyclosporine, is used if the patients are not responding to corticosteroids [6, 7]. Patients with MFC are at high risk for developing CNV, which occurs in up to 60% of cases. Different medications are used based on the classification of the lesions. The determination of the nature of the lesions is a key factor guiding clinical treatment. In the past, the activity of MFC lesions was usually determined by the changes of visual acuity, observation of vitreous inflammatory cells, and focal pigmentation [8] However, these indicators are lack of standard criteria and accuracy for clinicians to depend on. The clinicians have stopped the medication prematurely or given misdiagnosis when MFC reoccured [8]. Hence, different instrumental imaging techniques are vastly and periodically applied to examine, monitor, determine and classify the MFC lesions. These imageological techniques include slit-lamp microscope, indirect ophthalmoscope after dilated pupils, fundus color photography, infrared fundus photography, fundus auto fluorescence (FAF), fluorescein fundus angiography (FFA), and optical coherence tomography (OCT). The independent application of each of the technique provides diagnostic evidence for one or more types of MFC. The comprehensive application of these methods can determine and classify the lesion types of MFC and improve the diagnosis precision.

The classification of the MFC lesions are divided into four types, including active inflammatory lesions, inactive inflammatory lesions, secondary active choroidal neovascularization (CNV) and secondary inactive CNV [8]. The accurate imageology examinations can provide a more objective and accurate basis for the diagnosis of the activity of the lesions. In this study, we performed a variety of imaging examinations on a group of MFC patients and summarized the imaging features of the four types of MFC in order to provide a more accurate and comprehensive basis for the determination of clinical treatment options.

## Methods

### Objects

Fifty-one eyes of 28 patients diagnosed with MFC in our hospital from January 2010 to March 2017 were included in the study. These patients consist of 10 males and 18 females aged 31 to 49 years (mean age 41.5 ± 0.8 years). All patients underwent various fundus imaging examinations to assist the diagnosis and determine the types of MFC. 23 patients had MFC on both eye whilst 5 had monocular disease. 19 patients went to hospital because of visual acuity with or without visual distortion; 5 patients went see the doctor due to the occlusion or visual field defect, and 4 visited with the sensation of the shadow in front of the eyes. All eyes are in compliance with MFC diagnostic criteria [2]. We exclude patients with tuberculosis, syphilis, human immunodeficiency virus and other diseases with systemic inflammation or infectious diseases, multiple transient white point syndrome, punctate choroidal choroiditis, shotgun-like chorioretinopathy, toxoplasma retinal choroiditis from this study.

### Methods

All of the patients received examinations of fundus color photography, infrared fundus photography, fundus FAF, fluorescein fundus angiography (FFA), and optical coherence tomography (OCT), respectively.

(1) The fundus color photography was performed with TOPCON fundus camera (TRC-5EX; TOPCON, Beijing, China). After mydriasis, the patients first took color fundus images, and focused on collecting fundus images of the lesions.

(2) The infrared fundus photography, FAF and FFA examinations were performed with HRD fundus angiography instrument (Heidelberg, Germany). The posterior pole color fundus image and infrared fundus image were taken after dilation as the first step of examination. 3–5 FAF scanning images were acquired continuously by 488 nm laser afterwards. The images were processed by Herdelberg Eye Explore software to generate FAF examination results. Subsequently, the patients were given an intravenous injection of 3 ml 15% sodium fluorescein. After 8 to 10 s, a filter was added to the camera and images of each quadrant were obtained. The images were obtained initially by continuous shooting, and then changed to intermittent shooting to obtain the FFA image. The angiographic process was divided into preretinal artery phase, arterial phase, venous phase, and advanced phase. Preretinal arterial phase was the stage when the central retinal artery has not been filled with fluorescein; the arterial phase was the stage when the retinal artery began to be filled with fluorescein to the venous filling; the venous phase was the stage when venous was filled; the advanced phase was the late stage when fluorescein subsides from the retina. In this procedure, hyperfluorescence was defined as the increase of fluorescein accumulation, and hypofluorescence was defined as the decrease or disappearance of fluorescein.

(3) The OCT examinations were performed using the Heidelberg Spectralis HRA OCT instrument (Heidelberg) with a scanning depth of 5 to 8 mm. Horizontal scanning was done with the lesion as the center to generate OCT images. Images with best quality and position were selected for labeled storage. After all examinations were completed, the color fundus images, the FFA images and the FAF images were compared and analyzed to assist the MFC diagnosis.

The MFC lesions were classified as active inflammatory lesions, inactive inflammatory lesions, and active choroidal neovascularization (CNV) secondary inflammatory lesions and insecondary active CNV according to literature reports [8] and comprehensive fundus imaging examinations mentioned above. Finally, imageology features of different types of multifocal choroiditis were summarized.

## Results

### Clinic results

As shown in Table 1, active inflammatory lesions were observed in 36 eyes, accounting for 70.6%, and 11 eyes of them are diagnosed with secondary active CNV. Inactive inflammatory lesions are found in 5 eyes (9.8%), and 2 of them are diagnosed with secondary inactive CNV. Coexistence of active and inactive inflammatory lesion was observed in 10 eyes (19.6%), including 1 secondary active CNV and 2 secondary inactive CNV.

**Table 1:** Clinic results

Lesion classification	Eyes	Proportion (%)	
Active inflammatory lesion	36	70.6	Including 11 eyes were secondary active CNV
Inactive inflammatory lesion	5	9.8	Including 2 eyes were secondary inactive CNV
Coexistence of active and inactive inflammatory lesion	10	19.6	Including 1 eye was secondary active CNV, and 2 eyes were secondary inactive CNV

### Fundus color photography examination results

**Fig. 1:**
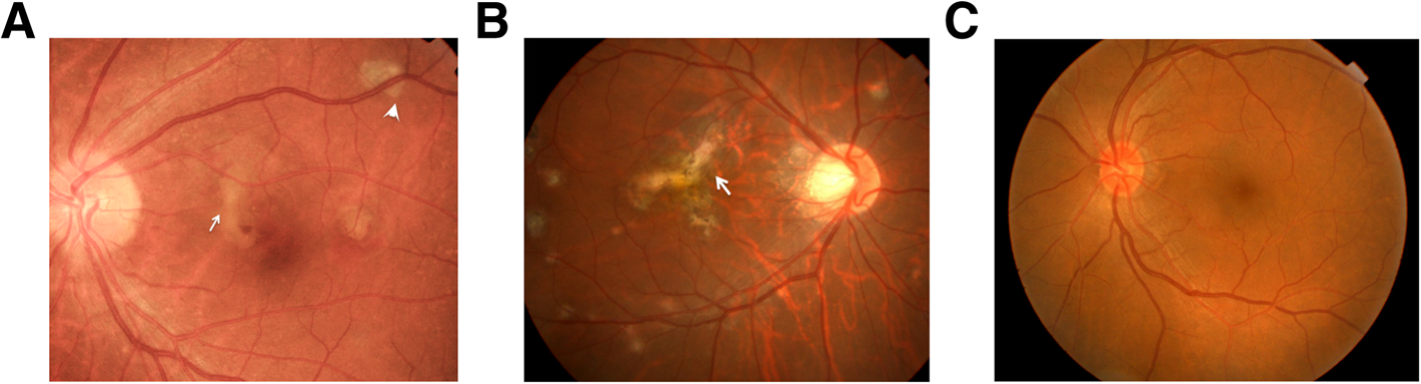
Typical fundus color photography images of the MFC eyes. **a** An eye where active inflammatory lesions and inactive inflammatory lesions were found coexists. Active inflammatory lesions are seen on the nasal side of the macula. They were yellowish white plaques with blurred edges and punctate hemorrhage (white arrow). An inactive inflammatory lesion was seen in the upper vascular arch, which was grayish white with clear borders (white arrow). **b** An eye where inactive inflammatory lesions were detected. Scattered old lesions with clear circular borders are located at the posterior pole. A scarred CNV can be seen in the macular area with pigmentation (white arrow). No bleeding and other lesions are found on this CNV. **c** The healthy control

The active inflammatory lesions are round or oval with diameters ranging from 45 to 350 μm in fundus color photography examinations. Most of them are isolated yellowish white lesions. The lesion boundaries with obvious inflammation are blurred due to retinal edema. Congested optic discs and blurred boundary lesions are found in 12 eyes with active inflammatory lesions. The border of inactive inflammatory lesions is clear and the color is grayish. The obvious pigmentation and typical perforation-like scar can be found on eyes with long course of the disease. Secondary active CNV has mild elevation of the lesion with retinal edema around. Bleeding in within the lesions can be detected in 4 eyes with secondary active CNV (Fig. 1a). There is no retinal edema exudation in the secondary inactive CNV. Scar fibrosis and different degrees of pigmentation can be detected on the CNV lesions (Fig. 1b).

### Infrared fundus photography examination results

**Fig. 2:**
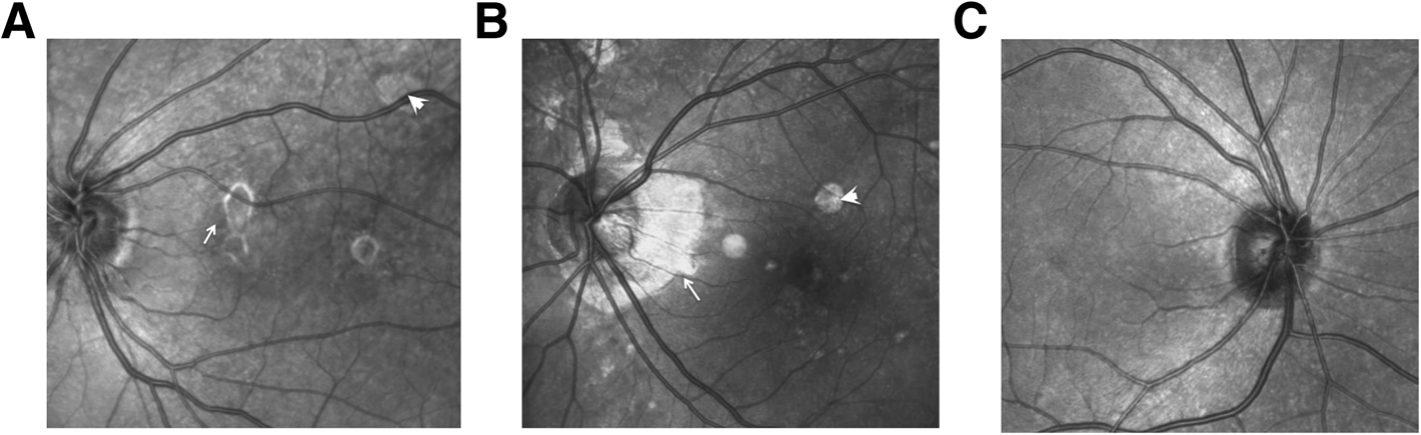
Typical infrared images of the MFC eyes. **a** The active inflammatory lesions seen on the nasal side of the macula is found to be homogeneous and weakly fluorescent with a hyperfluorescent boundary (white arrow). The fluorescence of the inactive lesion at the supraorbital vascular arch is homogeneous and strong (white arrow). **b** An eye where inactive inflammatory lesions were detected. The ring-shaped atrophy lesions near the disc are strongly fluorescent (white arrows). The multiple inactive inflammatory lesions in the posterior pole are detected as homogeneous and hyperfluorescence (white arrow). **c** The healthy control

Both active and inactive lesions exhibit as homogeneous punctate or flaky fluorescence in infrared fundus photography examinations (Fig. 2). The fluorescence of the CNV secondary lesions was uneven with brighter fluorescent borders (Fig. 2a).

### FAF examination results

**Fig. 3:**
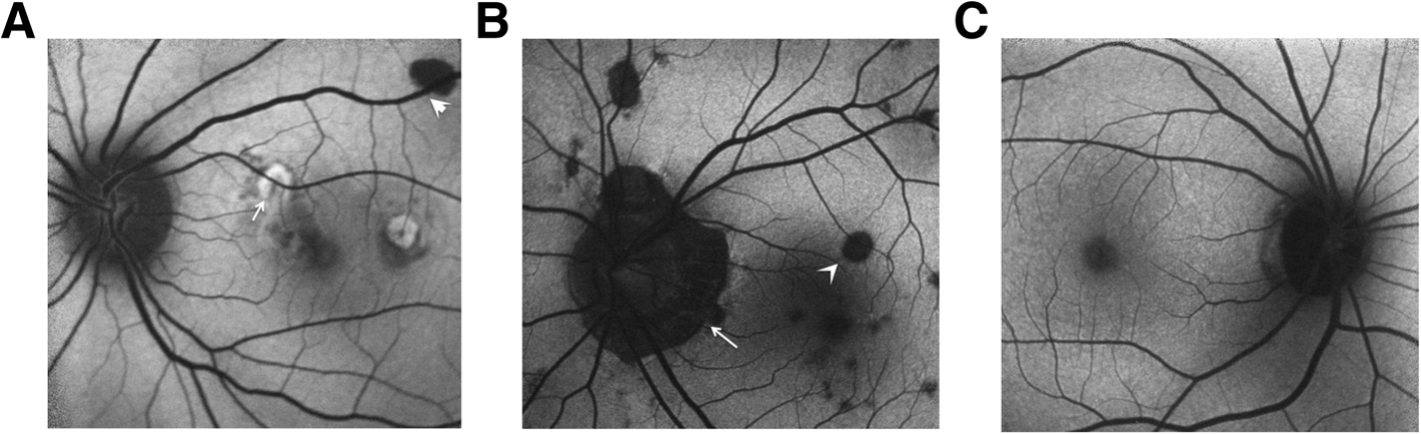
Typical FAF images of the MFC eyes. **a** The active inflammatory lesions on the nasal side of the macula exhibits irregular hyperautofluorescence. Part of the fluorescence is obscured by the bleeding around the lesion (white arrow). The inactive inflammatory lesion at the supraorbital vascular arch indicates as homogeneous hypoautofluorescence (white arrow). **b** The weakly fluorescent near the optic disc is the atrophy lesion (white arrow), and the uneven hypofluorescence in the posterior pole are the multiple inactive inflammatory lesions (white arrow). **c** The healthy control

In the FAF examinations, the active inflammatory showed low auto fluorescence (AF) at the center of the lesion with a strong fluorescence ring (Fig. 3a). Inactive inflammatory lesions were characterized by AF deficiency which exhibit as dark spots (Fig. 3b). The secondary active CNV showed strong AF with a stronger AF ring. The AF of the secondary active CNV could be veiled by the edema to be obscuration (Fig. 3a). The CNV secondary invasive lesion showed strong AF with a low AF ring (Fig. 3b).

**Fig. 4:**
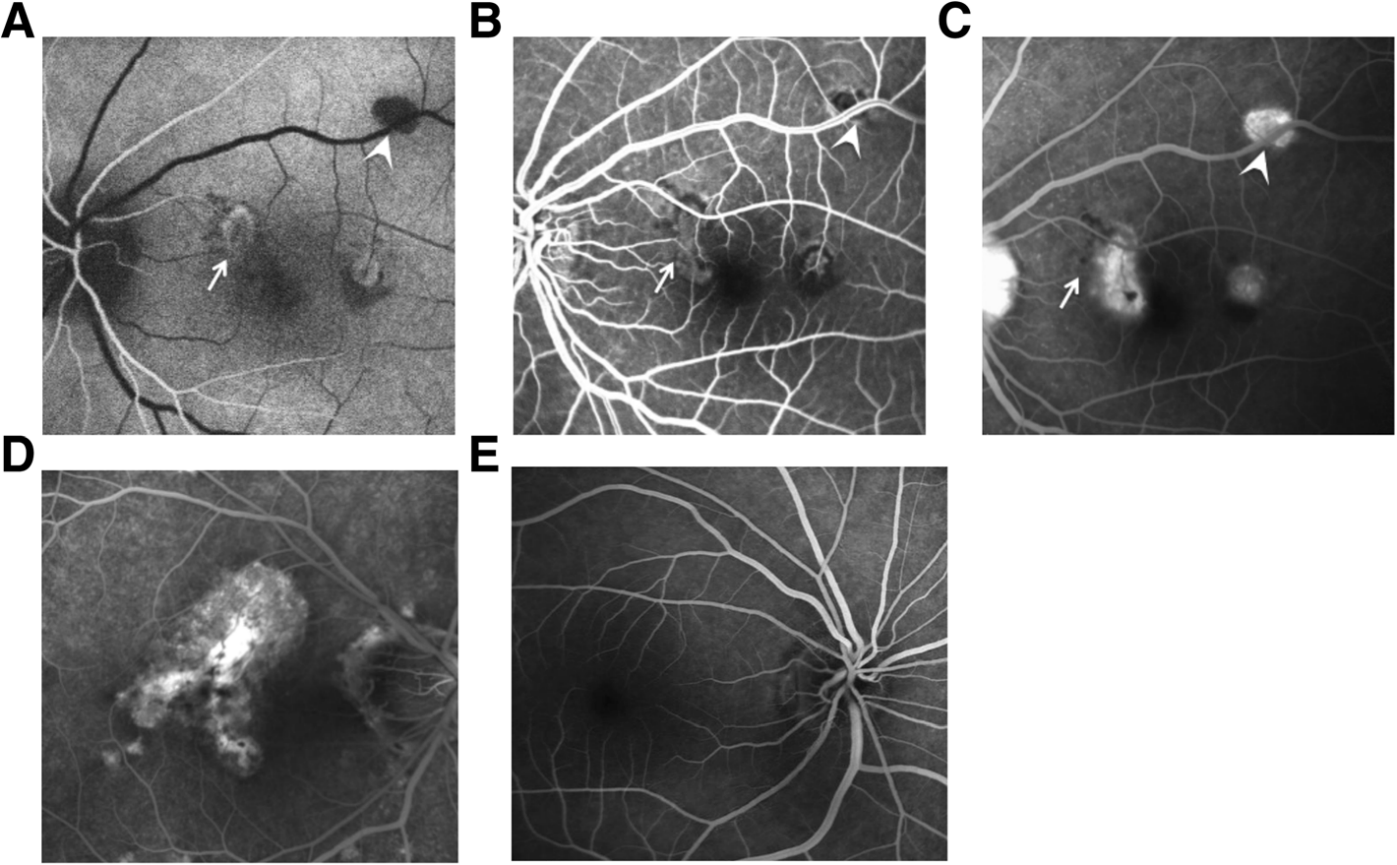
Typical FFA image of the MFC eyes. **a** The same eye in arterial stage. The active inflammatory lesion on the nasal side of the macula exhibits hyperfluorescence surrounding by a hypofluorescent boundary (white arrow). The inactive inflammatory lesion at the supraorbital vascular arch exhibits as a weakly fluorescent plaque (white arrow). **b** The same eye in venous stage. The active inflammatory lesions on the nasal side of the macula gradually increases, but the internal hemorrhage obscures the fluorescence (white arrow). The hypofluorescent rings around the inactive inflammatory lesion at the supraorbital vascular arch gradually strengthen in the FFA procedure (white arrow). **c** The same eye in advanced stage. The fluorescein leakage can be seen around the active inflammatory lesions on the nasal side of the macula with a blurred boundary. The fluorescence is obscured by the internal bleeding (white arrow). The inactive inflammatory lesion at the supraorbital vascular arch is homogeneously stained with optic disc leakage (white arrow). **d** The same eye in advanced stage. The atrophy lesions at the posterior pole and the optic disc are transflective. The CNV secondary inactive lesion at the macular area exhibits typical scar staining. **e** The healthy control

The active inflammatory lesions exhibit low AF in the early stage of FFA examination. The fluorescence gradually increases in the following stage with slight leakage. Inactive inflammatory lesions are typically transflective. The CNV morphology can be clearly seen in the early stage of the examination in the eyes with secondary active CNV. Obious CNV fluorescein leakage can be detected as the angiographic process progresses. The secondary inactive CNV exhibits scar staining in the FFA images (Fig. 4).

### OCT examination results

**Fig. 5:**
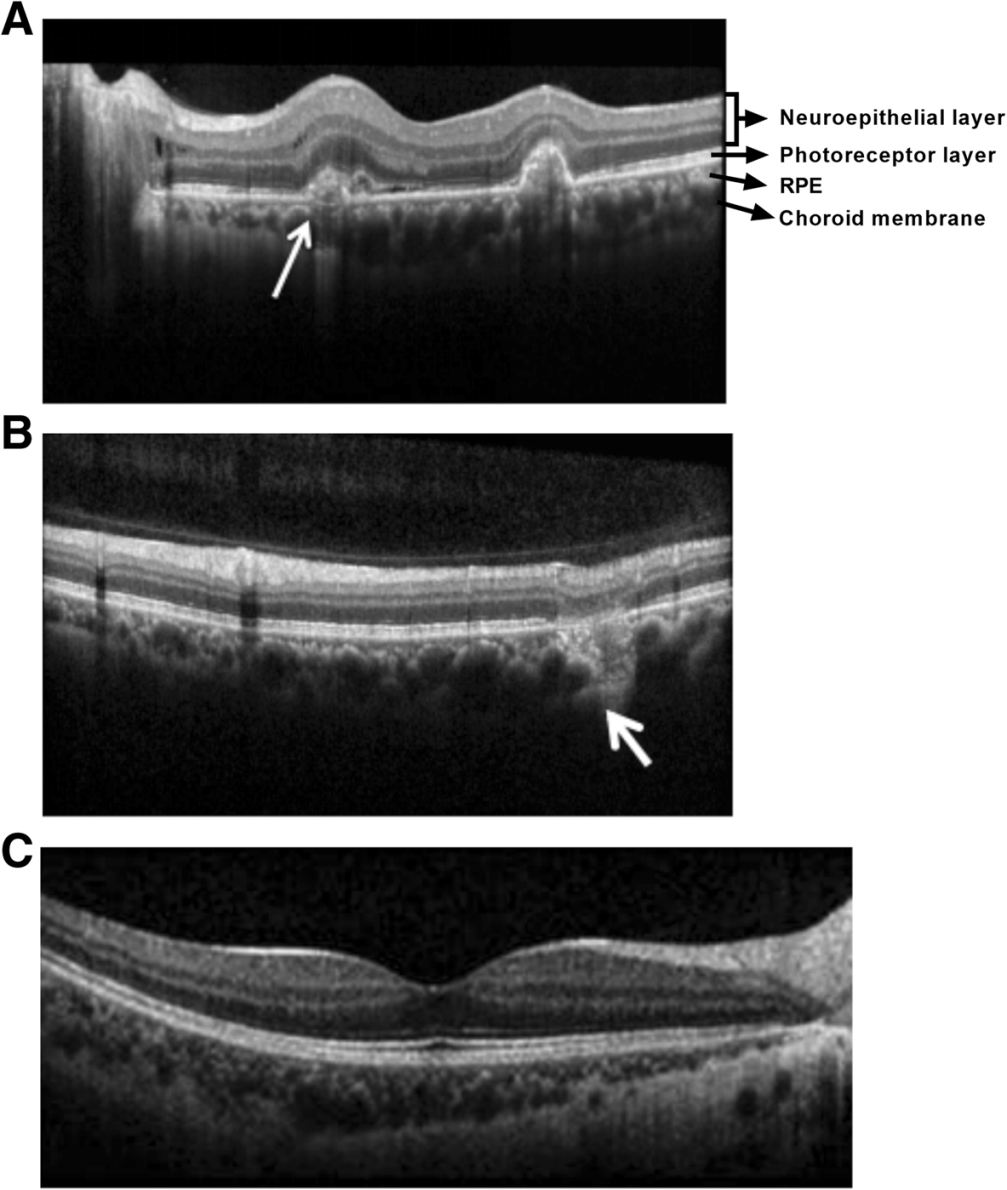
Typical OCT images of the MFC eyes. **a** The CNV secondary active inflammation lesion at the macula nasal elevates subretinally with a rough border and a few subretinal effusion (white arrow). **b** RPE-deficiency and choroidal scar formation of the inactive inflammatory lesion can be detected at the supraorbital vascular arch (white arrow). **c** The healthy control

OCT examinations indicate that, in the 36 eyes of active inflammatory lesions, 23 eyes have hyperreflection signals of RPE and choroidal while the conical or dome-like lesions underneath have hyporeflective signal with ellipsoidal structural damage (Fig. 5 a). Inactive inflammatory lesions exhibit as perforation-like RPE defects and choroidal scars (Fig. 5a), or as clear reflective signals of RPE elevation lesions. Retention of subretinal fluid can be seen on CNV secondary active inflammations (Fig. 5a), while RPE defects and choroid scar formation are seen in eyes with secondary CNV (Fig. 5b).

## Discussion

The composition of MFC lesions is complex in the eyes, especially for patients with long-term disease and repeated inflammation. The fundus lesions include active inflammation lesions, inactive inflammation lesions, and secondary active CNV, secondary inactive CNV [8]. Due to the complexity of the MFC lesions, single optical examination method is not sufficient for the detection and determination of the lesions. For example, active inflammation and secondary active CNV may be found fluorescein leakage on FFA examination. A comprehensive and in-depth understanding of the pathophysiological process of the MFC lesion development can significantly improve the accuracy of assessing the imaging features of the lesions, and further provide guidance to clinical treatment. In this study, the number of cases was limited, and the characteristic analysis of various stages of lesion was not very comprehensive. Thus, we reviewed relevant literature and combined our observations to analyze the multi-mode imaging features of various types of lesions in the process of lesion development. The discussion is as follows:

Inflammatory lesions initiate from the RPE and choroidal capillary levels. When a lesion further destroys the integrity of the RPE and Bruch membranes to provide a pathway for the growth of CNV, it becomes a CNV secondary lesion. It is reported that the proportion of CNV secondary lesions is higher in MFC than in other types of retinal choroiditis [8]. Corresponding to the pathophysiological process of MCP, different imaging examinations have different clinical significance, advantages and disadvantages in different stages of lesions.

The main function of fundus color photography is to observe the size, extent, optic disc and macular involvement of the lesion, and to judge the activity of the lesion according to the clearness of the lesion boundary, the combination of retinal edema and hemorrhage, and the formation of pigments in the lesion. However, it is difficult to provide evidence for the diagnosis of the re-active inflammatory lesions in the old lesions based solely on fundus color photography [9]. Infrared fundus photography cannot judge the activity of lesions, but the performance of infrared photography is different between simple inflammatory lesions and secondary inflammatory lesions of CNV. In addition, infrared photography can detect the lesions which cannot be shown by fundus color photography or FAF. Thus, infrared fundus photography has higher sensitivity.

The main function of FAF includes judging the function of RPE [10]. The mild inflammatory without RPE damage exhibits normal auto fluorescence. When the RPE is completely damaged by inflammation and the lipofuscin disappears, the lesion shows low auto fluorescence. Thus, the intensity of AF reflects the severity of lesion damage to some extent. In addition, the level of spontaneous fluorescence around the lesion is the major indicator for determining the activity of the lesions. Active inflammatory lesions show low AF in the interior, but surrounded by high AF, which confirms that the lesion was in the active stage and RPE was involved around the lesion. When the lesions enter the inactive stage, due to the damage of RPE, most of the lesions show the absence of AF, and the high AF around the lesion weakens and disappears due to RPE damage. But, the AF in the unaffected area is normal. When combined CNV is formed, CNV shows high AF inside the lesion. However, AF is insufficient in judging the activity of CNV [10].

The fluorescein leakage in FFA is the major indicator for determining the activity status of the lesions [11]. Active inflammatory lesions show mild strong fluorescence in the early stage and slight fluorescein leakage in the late stage, while non-active inflammatory lesions show typical pane-like defects and mild scar staining in the late stage. CNV morphology was observed in the early stage of active CNV angiography. In the advance stage, fluorescein leakage is faster and more significant than simple inflammatory lesion leakage [12]. This feature can provide imaging basis for clinicians to choose anti-VEGF therapy or anti-inflammatory therapy.

The main function of OCT is to judge whether CNV is secondary or not, whether retinal edema, retinal exudation changes and other activity indicators are involved. The most significant OCT characteristic of active lesions is the retention of subretinal fluid. However, Brydak-Godowska et al. reported that only 66.7% lesions have such exudative manifestations, and the remaining 33.3% of lesions do not have subretinal fluid retention [13]. Therefore, it is necessary to judge by combining the leakage of visible fluorescein in FFA. In addition, in OCT, both inflammatory lesions and CNV can be manifested as bulging lesions. What distinguishes them is the invasion of the RPE. CNV generally breaks through the RPE into the subretinal, and inflammation is more underneath [14]. OCT can also monitor the lesion development and recovery procedure of MFC by detecting the integrity of ellipsoidal band and RPE level. The structure of ellipsoidal band and RPE became gradually smoother in patients who enter the recovery phase [15].

It is worth noting that, one single traditional imaging method cannot completely distinguish all kinds of lesions. For example, active inflammatory lesions and secondary active CNV both may have fluorescein leakage in FFA and may exhibit as RPE lesions and prominent lesions on OCT. New imaging method such as OCT angiography, can detect whether there is a CNV component inside the lesion [16]. It suggests that single examination method is not sufficient for the comprehensive and accurate detection of all types of the lesions. Comprehensive application of different fundus imaging methods is necessary to generate a deeper understanding of MFC, and to make an accurate diagnosis to guide clinical treatment.

## Conclusion

Different MFC lesions exhibit different symptoms such as inflammation, internal bleeding, invasion of the RPE and choroidal scar. These symptoms can be detected by comprehensive optical examinations. We summarize the typical features of the lesions on fundus color photography, fundus autofluorescence, fluorescein fundus angiography, and optical coherence tomography as a guide for clinical diagnosis and therapy of MFC.

## Abbreviations

CNV: Choroidal neovascularization
FAF: Fundus autofluorescence
FFA: Fluorescein fundus angiography
MFC: Multifocal choroiditis
OCT: Optical coherence tomography
RPE: Retinal pigment epithelium
